# Metal(loid)s Spatial Distribution, Accumulation, and Potential Health Risk Assessment in Soil-Wheat Systems near a Pb/Zn Smelter in Henan Province, Central China

**DOI:** 10.3390/ijerph19052527

**Published:** 2022-02-22

**Authors:** Ling Yang, Qiang Ren, Shiji Ge, Zhiqiang Jiao, Wenhao Zhan, Runxiao Hou, Xinling Ruan, Yanfang Pan, Yangyang Wang

**Affiliations:** 1 National Demonstration Center for Environmental and Planning, College of Geography and Environmental Science, Henan University, Kaifeng 475004, China; yangling0606@163.com (L.Y.); 0412420xx@163.com (X.R.); 2 Key Laboratory of Geospatial Technology for the Middle and Lower Yellow River Regions, Henan University, Ministry of Education, Kaifeng 475004, China; 1610131137@vip.henu.edu.cn (Q.R.); GSGe@henu.edu.cn (S.G.); 3 Henan Engineering Research Center for Control and Remediation of Soil Heavy Metal Pollution, Henan University, Kaifeng 475004, China; ZQJiao@henu.edu.cn (Z.J.); hou20010423@163.com (R.H.); 4 National Key Laboratory of Human Factors Engineering, China Astronaut Research and Training Center, Beijing 100094, China; zhanwenhao2005@126.com

**Keywords:** health risk assessment, metal(loid)s, soil, spatial distribution, wheat

## Abstract

To understand the influence of Pb/Zn smelter on surrounding environment, 110 soil and 62 wheat grain samples (62 paired samples) were collected nearby a Pb/Zn smelter in Jiaozuo City, Henan Province, China. The content and spatial distribution of metal(loid)s in the soil-wheat system, and the potential health risk via consumption of wheat grains were determined. Results showed that the average content of Pb, Cd, As, Cu, Zn, and Ni in soil were 129.16, 4.28, 17.95, 20.43, 79.36, and 9.42 mg/kg, respectively. The content of Cd in almost all soil samples (99.1%) exceeded the national limitation of China (0.6 mg/kg). Spatial distribution analysis indicated that atmospheric deposition might be the main pollution source of Pb, Cd, As, and Zn in soil. In addition, the average content of Pb, Cd, As, Cu, Zn, and Ni in wheat grain were 0.62, 0.35, 0.10, 3.7, 35.77, and 0.15 mg/kg, respectively, with the average Pb and Cd content exceeding the national limitation of China. The average bioaccumulation factor of these metal(loid)s followed the following order: Zn (0.507) > Cu (0.239) > Cd (0.134) > Ni (0.024) > Pb (0.007) > As (0.006). Health risk assessment indicated that the average noncarcinogenic risk of children (6.78) was much higher than that of adults (2.83), and the carcinogenic risk of almost all wheat grain is higher than the acceptable range, with an average value of 2.43 × 10^−2^. These results indicated that humans who regularly consume these wheat grains might have a serious risk of noncarcinogenic and carcinogenic diseases.

## 1. Introduction

Rapid urbanization, agricultural modernization, and industrialization in the last few decades have resulted in a large amount of metal(loid)s released into the environment [1]. As the primary environmental reservoir and storage bank of metal(loid)s, soil accumulates most of the released metal(loid)s and is severely contaminated in some areas [2,3]. Based on the national survey of China in 2014, more than 10% of the farmland was contaminated with metal(loid)s [4,5], which was more serious in other developing countries. Metal(loid)s are toxic, nondegradable, and pose a serious risk to human health through the food chain bioaccumulation, which gained great attention worldwide [6]. A main pollution source of metal(loid)s is the nonferrous smelting and processing industry (NSPI), which can discharge metal(loid)s through the wastewater, atmospheric deposition, and solid waste [7,8]. Therefore, a study on the pollution status of metal(loid)s in the soil around these enterprises can provide an important guide for the safe use of land resources and control of human behavior.

Great attention has been given to the impact of NSPI on the surrounding soil in recent years [9,10,11,12]. Shen et al. [13] reported that the topsoil in the smelter area was highly contaminated by metals, and Cd and Zn mainly exist in mobile fractions, which can migrate even to a depth of 80 cm. A similar result has also been reported by Aminiyan et al. [9], indicating that copper smelter results in the surrounding soil were severely contaminated with metal(loid)s (As, Cd, Cr, Cu, Pb, and Zn), and these metal(loid)s were dramatically mobile and available. The metal(loid)s discharged by NSPI may be more active than that of other pollution sources, which may enter into the food chain easily and threaten the health of the ecosystem [14,15]. However, the attention given to the migration of metal(loid)s in the soil-crop system around NSPI is not enough.

The accumulation and translocation of metal(loid)s in a soil-maize system around a zinc-smelting area (southwest of China) has been reported by Duan et al. [16]. Their results indicated that targeted metal(loid)s in soil were significantly higher than the background values but had no potential noncarcinogenic risk for local maize consumers (adults and children), which may mainly attribute to the weak metal(loid)s accumulation capacity of maize [17]. In contrast, the metal(loid)s content in vegetables, rice grain, and fish around a large Cu-smelter all exceeded the maximum permissible level of food in China [18]. In addition, the metal(loid)s contamination in soil-crop systems around the NSPI has also been reported in several other countries [19,20]. However, there is little information about the migration of metal(loid)s in the soil-wheat system around the NSPI in China.

Henan province, located in the central-eastern region of China, is a major wheat-producing province, which contributes more than 27% of the total wheat production in China [21]. At the same time, NSPI is very developed in Henan province, especially for Pb/Zn smelting, which may result in metal(loid)s contamination in surrounding farmland [22,23]. In addition, wheat is generally considered a potential accumulator of metal(loid)s [17,24]. The wheat planted close to the smelter may accumulate various metal(loid)s and cause great risk to wheat consumers. Therefore, it is urgent to understand to what extent these metal(loid)s accumulated in soils and wheat grain around the NSPI. This study aimed to investigate: (1) the content and spatial distribution of metal(loid)s (Pb, Cd, As, Cu, Zn, and Ni) in the soil around a Pb/Zn smelter in Henan province; (2) the accumulation of these metal(loid)s in wheat grains; and (3) the potential health risk of these metal(loid)s to local wheat consumers.

## 2. Materials and Methods

### 2.1. Study Area and Samples Collection

**Figure 1:**
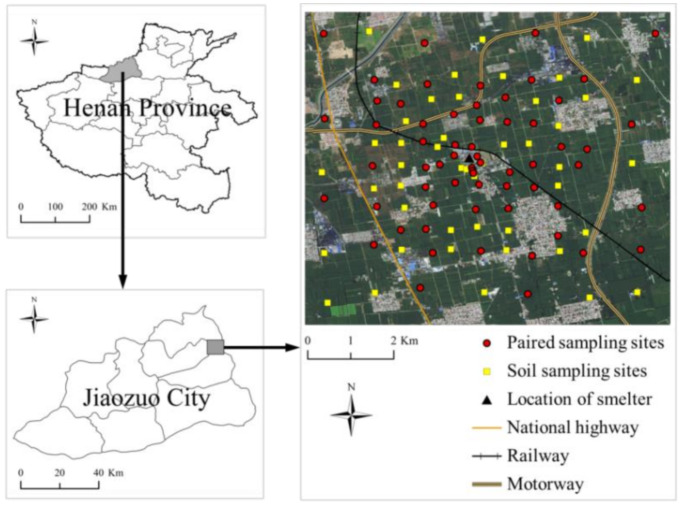
The study area and sampling sites.

The study area (around Dongfang Gold Lead Co., Ltd.: Jiaozuo, China) was located in the northwest of Henan province (Jiaozuo city), Huanghuai Plain, China (33°19′17.61″ N, 113°02.17″ E) (Figure 1). The climate of Jiaozuo City is a typical continental monsoon climate with four clearly distinct seasons. The annual average temperature and the annual precipitation are 14.9 °C and 603.5 mm. The soil in the study area was classified as Luvisol [25]. Jiaozuo is rich in mineral resources, including coal, bauxite, and pyrite. Sufficient energy supply promoted the rapid development of the nonferrous smelting industry, such as Pb/Zn smelter and aluminum smelting. Dongfang Gold Lead Co., Ltd. is one of the Pb/Zn smelting enterprises established in 2003; their main products are electrolytic lead, silver, gold, zinc oxide, copper, and other nonferrous metals. Due to inadequate environmental management, the company discharged a large amount of pollutants into the surrounding environment and was closed by the local government in 2014. After that, wheat and other crops were planted very close to the smelter by local farmers.

One hundred and ten soil and 62 wheat grain samples (62 paired samples) were collected around 36 km^2^ of Dongfang Gold Lead Co., Ltd. Approximately 500 g of surface soil samples (0–25 cm) were collected from each site, and soil was sampled from five individual locations at each site to minimize the heterogeneity. The collected soil samples were stored in polyethylene bags at room temperature, transported to the laboratory, air-dried and crushed (1 mm and 150 μm) for soil properties (pH, cation exchange capacity (CEC), organic matter content (OM), and total nitrogen (TN)) and metal(loid)s content detection. At the same time, 62 corresponding wheat ears samples (each location providing 30 wheat ears totaling 150 wheat ears per site) were collected and husked manually to obtain wheat grain. The wheat grains were washed with deionized water and dried to constant weight at 70 °C, then crushed into a fine powder with a stainless steel crusher (2 mm) for further analysis.

### 2.2. Chemical Analysis

The pH of the soil was determined by a pH meter (PHSJ-3F, INESA, Shanghai, China) with a soil-to-water ratio of 1:2.5 (*w*/*v*). Sodium acetate exchange and flame photometer (FP6410, INESA, Shanghai, China) were used to determine the CEC of these soil samples. OM content was determined by potassium dichromate oxidation and ferrous sulfate volumetric method (NY/T 1121.6-2006). TN was determined by an automatic Kjeldahl nitrogen analyzer (NKB3000, Hanon, Shanghai, China). The contents of metals (Pb, Cd, Cu, Zn, and Ni) in soil and wheat grain were measured by inductively coupled plasma mass spectrometry (ICP-MS, Thermo Fisher X2, Waltham, NA, USA) after digestion with an acid mixture [26]. For soil, 0.1 g of soil samples were digested in Teflon vessels with an acid mixture of HNO_3_ (9 mL)-HF (3 mL)-HClO_4_ (1.5 mL) at 180 °C until the solution became transparent, then transferred to a 50 mL volumetric flask and constant to volume with nitric acid (5%, *w*/*w*). For wheat grain, 0.5 g of wheat powder were digested with an acid mixture of HNO_3_ (12 mL)-HClO_4_ (2 mL) at 160 °C until the solution became transparent, then transferred to a 100 mL volumetric flask and constant to volume with nitric acid (5%, *w*/*w*). Arsenic (As) content in soil and wheat grain was determined by atomic fluorescence spectroscopy (AFS-3100, Haiguang Corp., Beijing, China) according to the description of Yu et al. [27].

### 2.3. Bioaccumulation Factor

Bioaccumulation factor (BF) was used to determine the transfer of metal(loid)s from soil to wheat grain. The BF was calculated based on the following Equation [26]:

(1) $$BF=\frac{C_{wheat}}{C_{soil}}$$

where *C_wheat_* represents the metal(loid)s content in wheat grain (mg/kg), *C_soil_* represents the metal(loid)s content in the corresponding soil sample (mg/kg). *C_wheat_* and *C_soil_* used for the *BF* calculation were the 62 paired samples, which were collected from the same point with the same coordinates.

### 2.4. Potential Health Risk Assessment

The noncarcinogenic risk of metal(loid)s to local residents via wheat consumption was assessed using the target hazard quotient (*HQ*) and hazard index (*HI*) according to the description of Liu et al. [28], which calculated by the following Equations:

(2) $$EDI=\frac{C_{i}\times IR\times EF\times ED}{BW\times AT}$$

(3) $$HQ=\frac{EDI}{RfD}$$

(4) $$HI=\sum_{i=1}^{n}HQ_{i}$$

where estimated daily intake (*EDI*) is the average daily dose of the considered element via food ingestion (mg/kg/day); *C*_i_ is the content of considered metal(loid)s in wheat grain (mg/kg); intake reference (*IR*) is the daily intake of wheat grain, 94.47 and 172.87 g/day for children and adults, respectively [29]; *EF* is the exposure frequency (days/year), 365 days/year for both children and adults; *ED* is the exposure duration (year), 12 and 70 years are assumed for children and adults, respectively [30]; *BW* is the average body weight of wheat consumers (kg), 15 and 63.5 kg for children and adults, respectively [29]; *AT* is the average time of metal(loid)s exposure (day), calculated by 365 days/year × *ED*; *RfD* is the reference dose (mg/kg/day), 0.0035, 0.001, 0.0003, 0.04, 0.3 and 0.02 mg/kg/day for Pb, Cd, As, Cu, Zn, and Ni, respectively [28]. The value of *HQ* or *HI* higher than 1 was assumed to have adverse effects on human health.

The cancer risk (*CR*) was used to determine the probability of an individual developing cancer disease due to exposure to a carcinogenic substance [31]. If there are multiple carcinogenic substances, the CR of each substance is added together. The CR is calculated by the following Equation:

(5) CR=EDI×SF

where *SF* is the cancer slope factor, the SFs for Cd, As, Pb and Ni were 6.1, 1.5, 0.0085, and 0.84 mg/kg/day, respectively [29,32]. If the value of *CR*<10^−4^, it is assumed that the cancer risk for wheat consumers is in an acceptable range.

### 2.5. Quality control and Quality Assurance

The standard reference materials (GBW07413 for soil and GBW10011 for wheat grain) were used for quality assurance and quality control. The recoveries of considered elements were between 96.7% and 106.2%. The detection limit and practical limit were 0.009 ug/L and 0.03 ug/L for Pb, 0.0005 ug/L and 0.0017 ug/L for Cd, 0.009 ug/L and 0.03 ug/L for Cu, 0.15 ug/L and 0.5 ug/L for Zn, 0.009 ug/L and 0.03 ug/L for Ni, and 0.017 ug/L and 0.053 ug/L for As, respectively.

### 2.6. Statistical Analysis

All data are presented as the means ± standard deviations. The spatial distribution of metal(loid)s in soil was created by ArcGIS 10.6 software (ESRI, Redlands, USA). The Spearman correlation analysis was conducted by SPSS (ver. 25.0)(IBM, NY, USA). The box plot was drawn by origin 8.5 (OriginLab, Northampton, MD, USA).

## 3. Results and Discussion

### 3.1. Metal(loid)s Content and Selected Properties of Soil

The contents of metal(loid)s in these soil samples are ranging from 12.04 to 2259.20 mg/kg for Pb, 0.54 to 64.89 mg/kg for Cd, 5.91 to 102.31 mg/kg for As, 5.00 to 87.93 mg/kg for Cu, 27.71 to 480.47 mg/kg for Zn, and 0.25 to 20.90 mg/kg for Ni, with the average contents of 129.16, 4.28, 17.95, 20.43, 79.36 and 9.42 mg/kg, respectively (Table 1). The average contents of these metal(loid)s are all much higher than that of the local background value (except for Ni) and lower than the national limitation of China (except for Cd).

The average content of Cd in soil (4.28 mg/kg) exceeded the national limitation of China (0.6 mg/kg, GB 15618-2018) more than six times. The minimum Cd content (0.54 mg/kg) was close to the national limitation. In addition, more than 99.1% of these soil samples with Cd content exceeded the national limitation. The number of soil samples with Pb, As, and Zn contents higher than the national limitation just occupied 13.6%, 8.2%, and 0.9% of the total soil samples. Therefore, Cd is the most polluted metal in the study area, followed by Pb, As, and Zn. In addition, previous studies reported that Cd could be accumulated by wheat more easily than other crops [17,33]; the high content of Cd in soil may result in serious pollution of wheat grain planted in the study area.

The coefficient of variation (CV) of Pb and Cd in soil was higher than 200%, and the CVs of As, Cu, and Zn in the soil also exceeded 50%, indicating that these metal(loid)s differed greatly among different soil samples and mainly caused by anthropogenic activities [34]. This result is similar to many previous reports [17,35,36]. The study area of the present study is 36 km^2^, and just part of these soil samples was dramatically influenced by the Pb/Zn smelter, which may be the main reason for the high CV value.

The pH, OM, CEC, and TN of these soil samples ranged from 8.13 to 9.07, 2.30 to 63.20 g/kg, 12.46 to 32.31 cmol/kg, and 0.02 to 0.93 g/kg, respectively. The soil in the study area is alkaline, with an average pH of 8.57. Most previous studies have indicated that higher pH can reduce the activity of metals but increase the activity of As in soil [37,38]. Therefore, the risk of As contamination in wheat grain should be paid more attention to. The average OM content was 25.48 g/kg, which belongs to the moderate scale (20–30 g/kg) [39]. The CVs of pH, OM, and CEC were lower than 32%, indicating anthropogenic activities have little influence on these soil properties. The CV value of TN was 63.97%, which may be attributed to the different land-use types and fertilization patterns [40].

### 3.2. Spatial Distribution of Metal(loid)s

**Figure 2:**
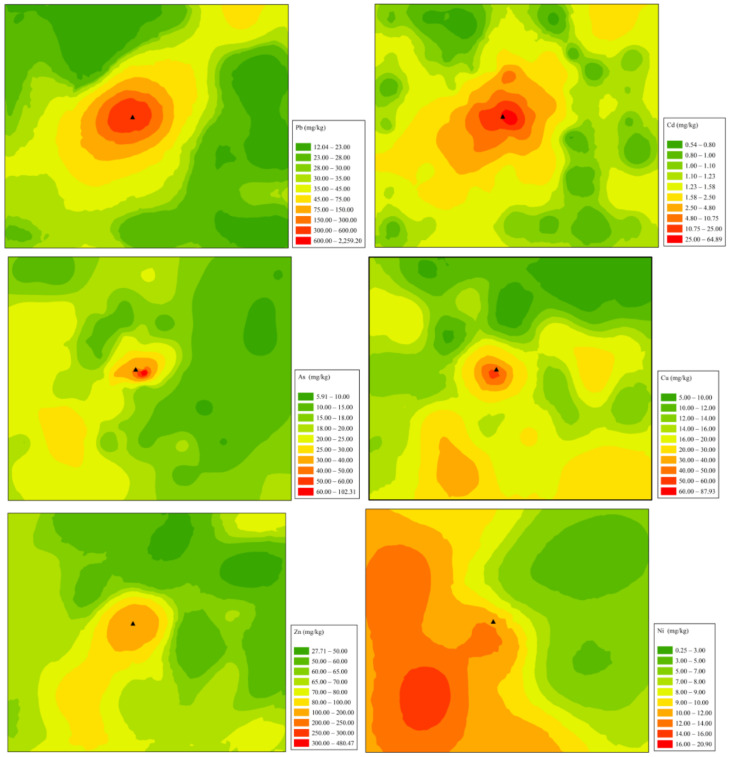
Spatial distribution of metal(loid)s in soil around the Pb/Zn smelter. ▲ Represents the location of the smelter.

The spatial distribution of these metal(loid)s in soil is shown in Figure 2. The distribution of Pb, Cd, As, and Zn are similar, and their contents decreased gradually with the increase of the distance far from the smelter. Field research indicated that there was no sewage irrigation and inappropriate disposal of the smelting solid waste in the study area in the past. Therefore, atmospheric deposition may be attributed to the main source for Pb, Cd, As, and Zn contamination in the soil near the smelter. In addition, the distribution of Pb, Cd, As, and Zn in the study area is oval from northeast to southwest, which is consistent with the local dominant wind direction (southwest wind) [41,42].

The content of Ni in soil was distributed in the band, which may mainly be caused by the highway (S233). Previous studies indicated that highways could cause metal(loid)s accumulation along with the soil, which is consistent with our present studies [43]. Besides the central area (very close to the smelter), there is still a high-value area of Cu in the southeast of the study area, which coincides with the position of a piggery. As a trace element of livestock, the average content of Cu in pig feeds reached 200–300 mg/kg [44,45], and most of this Cu can be excreted by urine and manure [46]. More importantly, pig manure has been widely used as organic fertilizers by farmers in China, which resulted in the accumulation of Cu in the soil near the piggery.

### 3.3. Metal(loid)s Content in Wheat Grain

The contents of metal(loid)s in wheat grain are ranging from 0.001 to 10.75 mg/kg for Pb, 0.04 to 1.73 mg/kg for Cd, 0.03 to 0.67 mg/kg for As, 1.55 to 5.7 mg/kg for Cu, 21.32 to 54.81 mg/kg for Zn, and 0.001 to 0.48 mg/kg for Ni, respectively (Table 2). The average contents of Cd and Pb in wheat grain are 0.35 and 0.62 mg/kg, which all exceeded the national limitation of China (0.1 mg/kg for Cd and 0.5 mg/kg for Pb, GB 2762-2017). The International Agency for Research on Cancer has classified Cd and Pb as Group I and Group II carcinogens, respectively, which can cause detrimental effects on the reproductive, immune system, nervous and cardiovascular [47,48]. Therefore, the high content of Cd and Pb in wheat grain may cause serious health risks to local residents via wheat consumption.

In fact, the content of Pb and Cd in soil exceeded the national limitation of China, occupying 13.6% and 99.1% of the total soil samples, respectively. Whereas, the proportions of Pb and Cd content in wheat grain exceeded the national limitation of China were 22.58% and 62.90%. Correlation analysis indicated that the correlation between the metal(loid)s content in wheat grain and soil properties was not significant (Table 3). In addition, metal(loid)s content in wheat grain (except for Cu) was significantly positively correlated with soil Pb and Cd. Furthermore, the wheat grain Pb content also positively correlated with soil As (*p* = 0.01) and Zn (*p* = 0.01), the wheat grain Cd content also positively correlated with soil As (*p* = 0.05), Cu (*p* = 0.01) and Zn (*p* = 0.01), the wheat grain As and Zn content also positively correlated with soil Cu (*p* = 0.01) and Zn (*p* = 0.05) (Table 3). These results may imply that the metal(loid)s contents in wheat grain are mainly dependent on their total contents in soil and the influence of other factors (fraction of metals in soil, soil OM content, and soil pH) on the accumulation of metals in wheat grains is nonsignificant. This result is inconsistent with previous studies [49,50].

Although As content in part of the soil samples exceeded the national limitation of China, just one wheat grain sample with As content higher than 0.5 mg/kg, indicating that the activity of As in soil is low even the soil pH is alkaline. The average contents of Cu and Zn in wheat grain were 3.70 and 35.77 mg/kg, respectively (Table 2), which are lower than the national limitation of China (NY 861-2004). In addition, Cu and Zn are essential trace elements for humans [51]; a small amount of Cu and Zn intake by wheat may have a positive effect on human health. The average Ni content in wheat grain is 0.15 mg/kg, but its content in wheat grain was not limited in China. Previous reports have indicated that Ni is toxic and carcinogenic [51], the neglect of Ni in the national standards of China may be harmful to human health, which should be given more attention.

### 3.4. Bioaccumulation of Metal(loid)s in Wheat Grain

**Figure 3:**
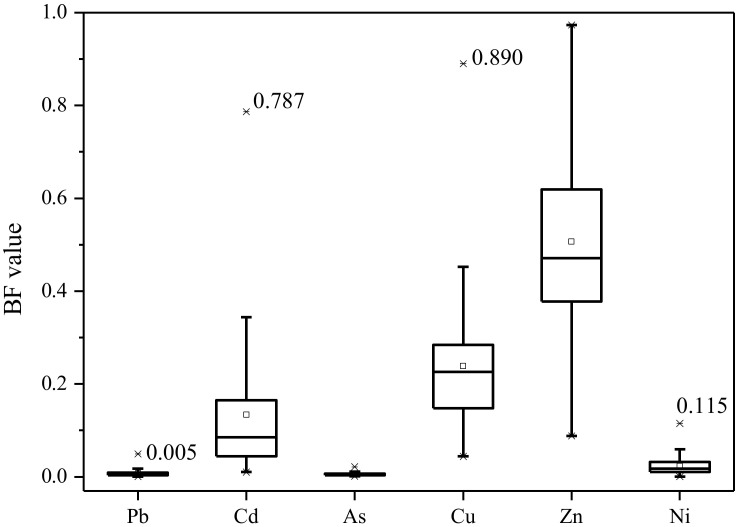
Bioaccumulation factor of metal(loid)s in wheat grain (*n* = 62).

The BF values of these metal(loid)s ranged from 0.001 to 0.050 for Pb, 0.010 to 0.787 for Cd, 0.006 to 0.022 for As, 0.239 to 0.890 for Cu, 0.507 to 0.973 for Zn, and 0.024 to 0.115 for Ni, respectively, with the average BF value decreased in the following order: Zn > Cu > Cd > Ni > Pb > As (Figure 3). Due to the high toxicity of Cd, most of the previous reports emphasized that Cd can be accumulated in wheat grain easier than other metals [17,30], and just a few attentions paid to the BF value of Cu and Zn in wheat.

The average BF values of Cu and Zn were 0.239 and 0.507, respectively, which are much higher than that of other metal(loid)s considered in the present study. Similar results have been reported in many previous studies [29,52]. Fortunately, Cu and Zn are essential trace elements for the metabolism of plants and mammals; the high BF value of Cu and Zn in wheat may be important for the health of mammals [53]. The average BF value of Cd was 0.134, which is much higher than that of Pb (0.007), As (0.006), and Ni (0.024). Previous reports indicated that the accumulation capacity of wheat to Cd might be the highest among various toxic metals [29,54], which are consistent well with the present study.

### 3.5. Potential Health Risk Assessment

**Figure 4:**
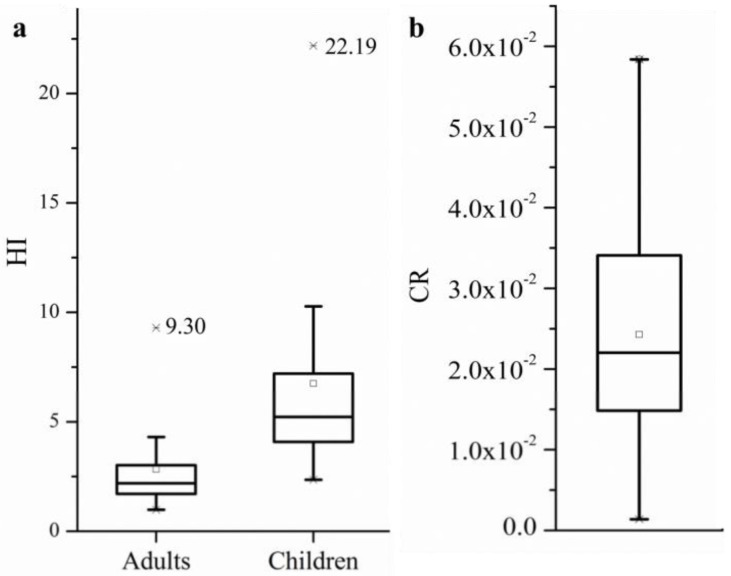
Potential health risk assessment of metal(loid)s for local wheat consumers. (**a**): noncarcinogenic risk assessment; (**b**): carcinogenic risk assessment.

The potential noncarcinogenic diseases risk index (HI) of metal(loid)s for local wheat consumers are shown in Figure 4a. The HI by intake of wheat grain ranged from 0.99 to 9.30 for adults and 2.35 to 22.19 for children, with an average HI of 2.83 and 6.75, respectively. The HI values of all wheat samples are higher than the acceptable range (HI<1) for both adults and children (except for one wheat sample with HI of 0.99), which indicates that local residents had a high risk of noncarcinogenic diseases due to the intake of metal(loid)s contaminated wheat [55,56]. Furthermore, part of the wheat samples with the HQ_Cd_ (19 and 29 for adults and children), HQ_As_ (20 and 54 for adults and children), HQ_Pb_ (5 for children), and HQ_Zn_ (6 for children) were higher than 1 (Table 4). Cd and As exposure was the main contributor of HI, with the average contribution higher than 63% for both adults and children.

The minimum and maximum CR values calculated based on Equation (5) are 1.39 × 10^−3^ and 5.84 × 10^−2^, respectively (Figure 4b). The CR values of all wheat samples are much higher than the acceptable range recommended by EPA (1.0 × 10^−4^) [26], indicating local wheat consumers are at serious risk of carcinogenic diseases. In addition, the minimum CR value of Cd, As, and Ni were 6.0 × 10^−3^, 4.5 × 10^−4^, and 2.4 × 10^−2^, respectively, which all exceeded the acceptable range (1.0 × 10^−4^). The sensitivity of human organs to different metal(loid)s is different [57], and the excessively multi-metal(loid)s exposure may result in various carcinogenic diseases to local wheat consumers.

In fact, studies regard to the influence of anthropogenic activities on metal(loid)s accumulation in wheat grain and related potential health risks have been carried out in many previous reports [28,29,31,56]. The long-term application of phosphorus fertilizer or nitrogen fertilizer increased the accumulation of metal(loid)s in wheat grain and the related potential health to humans, but their HI and CR values are still within the acceptable range [29,31]. However, sewage irrigation and artisanal mining can result in excessive accumulation of metal(loid)s in wheat and generate a serious threat to human health [56,58], which are consistent with our present studies. In addition, the Pb/Zn smelter result in serious metal(loid)s contamination in more than 36 km^2^ of both farmland soil and wheat in the present study. These results suggested that more attention should be paid to land use planning, which can reduce their influence on agricultural production and food safety.

This section may be divided into subheadings. It should provide a concise and precise description of the experimental results, their interpretation, as well as the experimental conclusions that can be drawn.

## 4. Conclusions

In conclusion, Pb/Zn smelting results in the surrounding soil being seriously contaminated with metal(loid)s, especially for Cd. The distribution of these metal(loid)s differ greatly and may be caused by a different source. The accumulation capacity of Cu and Zn was much higher than other elements, but their content in wheat grain was all within the national limitation of China, whereas the average contents of Pb and Cd in wheat grain exceeded the national limitation of China. The accumulation of metal(loid)s in wheat grain generated serious health risks (both noncarcinogenic and carcinogenic risk) to local wheat consumers. These results suggested that more reasonable land-use planning should be established to reduce the impact of Pb/Zn smelting on the surrounding environment.

## Figures and Tables

**Table 1:** Descriptive statistic of metal(loid)s and selected soil properties (*n* = 110).

	pH	OM (g/kg)	CEC (cmol/kg)	TN (g/kg)	Pb (mg/kg)	Cd (mg/kg)	As (mg/kg)	Cu (mg/kg)	Zn (mg/kg)	Ni (mg/kg)
Min	8.13	2.30	12.46	0.02	12.04	0.54	5.91	5.00	27.71	0.25
Max	9.07	63.20	32.31	0.93	2259.20	64.89	102.31	87.93	480.47	20.90
Average	8.57	25.48	23.17	0.22	129.16	4.28	17.95	20.43	79.36	9.42
SD	0.16	8.15	4.58	0.14	296.40	8.72	10.43	14.83	50.60	4.03
CV (%)	1.9	31.97	19.77	63.97	229.47	203.74	58.13	72.56	63.76	42.77
Background value *	-	-	-	-	22.30	0.07	9.8	20.00	62.50	27.40
Risk screening values **	-	-	-	-	170	0.6	25	100	300	190

* China National Environmental Monitoring Centre. Background values of soil elements in China. China Environmental Science Press [M]. Beijing, China. ** The risk screening values are cited from the national standard of China (GB 15618-2018). SD: standard deviation; CV: coefficient of variation; OM: organic matter content; CEC: cation exchange capacity; TN: total nitrogen content.

**Table 2:** Descriptive statistic of metal(loid)s in wheat grain (*n* = 62).

	Pb (mg/kg)	Cd (mg/kg)	As (mg/kg)	Cu (mg/kg)	Zn (mg/kg)	Ni (mg/kg)
Min	0.001	0.04	0.03	1.55	21.32	0.001
Max	10.75	1.73	0.67	5.70	54.81	0.48
Average	0.62	0.35	0.11	3.70	35.77	0.15
SD	1.55	0.43	0.10	0.92	7.34	0.12
CV (%)	249.50	125.12	90.80	24.76	20.53	75.96
National limits	0.5 *	0.1^*^	0.5 *	10 **	50 **	-

* National standard of China on the maximum residue limits of pollutants in food (GB 2762-2017); ** Limits by Ministry of Agriculture of China (NY 861-2004); ‘-’ represent no limitation in the standard.

**Table 3:** Correlation analysis between metal(loid)s content in wheat grain with its content in soil and soil properties (*n* = 62).

	W-Pb	W-Cd	W-As	W-Cu	W-Zn	W-Ni
pH	−0.042	0.151	−0.095	−0.208	−0.016	−0.160
TN	−0.162	0.085	−0.003	−0.029	−0.057	−0.040
OM	−0.192	0.041	0.039	0.000	−0.100	−0.069
CEC	−0.037	0.136	−0.094	−0.211	0.071	0.066
S-Pb	0.810 **	0.631 **	0.452 **	−0.063	0.550 **	0.315 *
S-Cd	0.824 **	0.607 **	0.397 **	−0.018	0.534 **	0.295 *
S-As	0.416 **	0.263 *	0.069	0.143	0.168	−0.004
S-Cu	0.234	0.419 **	0.345 **	0.064	0.416 **	0.081
S-Zn	0.511 **	0.379 **	0.313 *	−0.100	0.319 *	0.138
S-Ni	0.135	0.192	−0.206	−0.150	0.125	−0.132

* significance level of 0.05; ** significance level of 0.01; TN: total nitrogen content; OM: organic matter content; CEC: cation exchange capacity; W-: the content of metal(loid)s in wheat grain; S-: the content of metal(loid)s in soil.

**Table 4:** Number of the HQ higher or lower than 1 for individual element in adults and children.

	Adults		Children	
	HQ>1	HQ<1	HQ>1	HQ<1
Pb	0	62	5	57
Cd	19	43	29	33
As	20	42	54	8
Cu	0	62	0	62
Zn	0	62	6	56
Ni	0	62	0	62

## Data Availability

Not applicable.
